# Allosteric Proteasome Activation Interactions Drive Tau Degradation Efficiency

**DOI:** 10.1002/alz70855_102534

**Published:** 2025-12-23

**Authors:** Bryan D. Ryder, Nicholas L. Yan, Jason E. Gestwicki, ALBORADA Drug Discovery Team

**Affiliations:** ^1^ University of California, San Francisco, San Francisco, CA, USA; ^2^ University of California San Francisco, San Francisco, CA, USA

## Abstract

**Background:**

When inserted onto the C‐terminus of archaeal proteasome activator PA26, an optimized activator peptide derived from human 19S (‐NLSYYT‐OH) has been shown to efficiently degrade tau *in vitro*. Structure‐activity relationship experiments show how small steric forces generated from the leucine in position 5 (P5) account for full 20S proteasome gate opening and efficient degradation of tau.

**Method:**

Proteasomal activity assays using short 6‐amino acids peptides derived from (‐NLSYYT) show activity dependency on the presence of leucine. After cloning select mutants P5F, P5Y, P5W, P5V, and P5I onto the C‐terminus of aPA26, fluorescence polarization and OpenSPR show these mutations impact PA26‐20S affinity as well as activity. Cryo‐EM structures of each aPA26 mutant reveal a series of intermediate states of the 20S gate, and how steric interactions between leucine and a conserved arginine on the 20S drive allosteric interactions to fully open the 20S gate. A combination of *in vitro* assays and cell‐based assays were used to track total tau and *p*‐tau degradation in a dose dependent manner.

**Result:**

Mutations to the P5 leucine in the optimized NLSYYT proteasome activator peptide led to a significant loss of proteasomal activity. Even biochemically similar amino acids showed significant loss of activity (40%‐90%). When this sequence was cloned onto PA26 for multivalent sequence activation of the 20S proteasome, we found that in addition to loss in activity, these mutations also weakened affinity to the 20S proteasome. Cryo‐EM structures revealed that steric interactions lift PSMA5 R20 and trigger a network of allosteric interactions to turn and open the 20S gate. For P5W or P5Y, intermediate structures show specific pockets where these mutations break the allosteric network, resulting in partial gate opening. Tau degradation experiments validated that these partial open states yield less efficient degradation compared to full gate opening.

**Conclusion:**

The sensitivity of small hydrophobic amino acid leucine in the P5 position of NLSYYT significantly impacts the stability of the 20S gate. Remaining protein‐protein interactions within a partially closed 20S gate clash with tau entry. By leveraging this information, proteasome activators designed for tau degradation strategies can be designed around controlling the rate of tau degradation.

